# Tumor risks and surveillance outcomes in *SDHA* variant carriers

**DOI:** 10.1530/EO-26-0048

**Published:** 2026-09-11

**Authors:** Kelsey Ellis, Daniel Christensen, Jacob Beiringer, Anne Naumer, Jennie Vagher, Richard Wiggins, Hilary McCrary, Luke Buchmann

**Affiliations:** ^1^ Inherited Cancer Research Shared Resource, Huntsman Cancer Institute, University of Utah, Salt Lake City, Utah, USA; ^2^ Department of Otolaryngology – Head and Neck Surgery, University of Utah, Salt Lake City, Utah, USA; ^3^ Department of Radiology, University of Utah, Salt Lake City, Utah, USA

**Keywords:** *SDHA*, succinate dehydrogenase, paraganglioma, pheochromocytoma, surveillance, genetic testing, hereditary cancer, gastrointestional stromal tumor (GIST)

## Abstract

**Objective:**

*SDHA* germline pathogenic variants confer a lower paraganglioma risk compared with other hereditary paraganglioma genes. Reduced screening for carriers without a personal or family history of *SDHA*-associated tumors has been proposed; however, supporting data remain limited. This single-institution cohort study addresses this by characterizing *SDHA* carrier tumor features and long-term screening outcomes.

**Design:**

This is a retrospective cohort study with longitudinal surveillance follow-up.

**Methods:**

Clinical records of patients with an *SDHA* likely pathogenic or pathogenic germline variant identified between July 2015 and July 2025 in the Family Cancer Assessment Clinic at the University of Utah Huntsman Cancer Institute were reviewed.

**Results:**

Among 106 *SDHA* carriers, 15% had a personal history of an *SDHA*-associated tumor. Of identified tumors, 47% were metastatic or demonstrated malignant behavior (3/3 extra-adrenal PPGLs, 4/5 GISTs, 0/7 head/neck PPGLs). No affected carriers had known family history of *SDHA*-associated tumors, and no cases of multiple related tumors were observed. Most carriers (63%) lacked a personal or family history of *SDHA*-associated tumors. Over 10 years of high-risk screening, zero previously unidentified *SDHA*-associated tumors were found.

**Conclusion:**

Our findings align with prior evidence that *SDHA* variants confer a lower tumor risk than other hereditary PPGL genes, lending support to an *SDHA*-specific screening approach. However, higher rates of malignant tumor behavior in affected carriers compared with sporadic disease suggest *SDHA* variants may be impactful in contexts like treatment decision-making. These contrasts highlight that while modifying or forgoing tumor screening may be reasonable for many carriers, a nuanced clinical approach remains essential for this population.

## Introduction

Pheochromocytomas and paragangliomas (PPGLs) are rare neuroendocrine tumors with a high degree of heritability. Up to 30–40% of individuals diagnosed with a PPGL are estimated to be carriers of a germline pathogenic or likely pathogenic variant (GPV) related to hereditary PPGL risk (1, 2). Well-established genes associated primarily with hereditary PPGL risk include the SDHx genes (*SDHA*, *SDHB*, *SDHC*, *SDHD*, *SDHAF2*), *TMEM127*, and *MAX*. GPVs in genes such as *VHL*, *FH*, *NF1*, and *RET* also have an increased PPGL risk as part of their tumor predisposition spectrum (2, 3). Unaffected carriers with a GPV that increases PPGL risk are typically recommended to undergo periodic screening with the goal of detecting any tumors at an early stage, so they can proceed with close surveillance or surgical resection (4, 5). This is in part because GPV carriers have a higher chance than individuals with a sporadic PPGL to develop metastatic disease or multiple tumors (1, 3, 6). The average age of onset is also lower than for a sporadic PPGL (1). In addition, these GPVs can increase the risk of other types of cancers: for example, SDHx GPVs are associated with increased wild-type gastrointestinal stromal tumor (GIST) and renal cell cancer (RCC) risks (6, 7, 8).

*SDHA* GPVs were first established as associated with hereditary PPGL risk in 2010, and *SDHA* analysis was widely available on clinical multigene panel testing by 2015. Studies have since demonstrated that *SDHA* GPVs have reduced penetrance compared with other hereditary PPGL predisposition genes (9, 10, 11). Recent data have documented lifetime PPGL risks of 0.1–10% for *SDHA* carriers, compared with penetrance for other well-described hereditary PPGL genes, such as *SDHB* (24–58%), *SDHC* (25%), and *SDHD* (43%) (7, 9, 10, 12). Penetrance of GISTs and RCCs is less well characterized in the existing literature but is thought to be lower than that of PPGLs (13, 14).

As multigene panel testing for hereditary cancer risk becomes more widespread, *SDHA* variants are a common secondary finding in carriers undergoing testing due to personal or family history of other cancers (1, 15, 16). While guidelines for other hereditary PPGL genes universally endorse increased PPGL screening for all GPV carriers, *SDHA*-specific guidelines have begun to emerge that recommend approaching screening for *SDHA* GPV carriers without a related personal or family history of tumors differently than other SDHx carriers (4, 5, 17).

For example, 2023 UK guidelines on screening for *SDHA* carriers do not recommend increased screening for *SDHA* carriers without a related personal or family history (17, 18). The National Comprehensive Cancer Network (NCCN) guidelines (v.3.2025) recommend consideration of modified screening intervals for *SDHA* carriers overall (4). The 2025 guidelines for hereditary PPGLs in the pediatric population have also moved toward a more nuanced approach to patient decision-making, although surveillance was still universally recommended for *SDHA* carriers due to ongoing limitations in data about this population (19). The lack of evidence demonstrating that family history of an *SDHA*-associated tumor is a reasonable proxy for increased personal tumor risk has been brought up by these guidelines and another recent commentary on screening for *SDHA* carriers as an indication that continued universal screening of *SDHA* carriers may be a reasonable option for the time being (19, 20).

For carriers with a GPV in *SDHA* or another SDHx gene undergoing screening, guidelines typically recommend annual biochemical testing via plasma or 24-h urine metanephrines alongside cross-sectional imaging of skull base to pelvis via PET/CT, WBMRI, or MRI/CT series every 2–3 years (5, 21). Biochemical testing identifies elevated metanephrine levels indicative of a functional or catecholamine-secreting PPGL, while imaging can detect both functional and non-functional PPGLs arising from the paraganglia from skull base down through the pelvis. This ongoing imaging has the potential to be both time- and cost-intensive for patients, particularly for carriers discovered to have a GPV in their childhood or teenage years who may have decades of screening ahead of them (5, 22). Continued evaluation of the effectiveness and utility of PPGL screening for *SDHA* carriers can help ensure this population is not being over- or underscreened.

Overall, data about long-term screening for *SDHA* carriers that may support *SDHA*-specific screening guidelines are limited. To make evidence-based decisions about screening for this population, further characterization of *SDHA* GPV is important to better understand tumor risks. Our analysis of a single-center cohort of *SDHA* GPV carriers seeks to expand on this body of evidence. Our multidisciplinary, high-risk paraganglioma clinic acts as a touchpoint for centralized follow-up for people with GPV in genes that increase PPGL risk, including *SDHA.* This long-term screening outcome data can facilitate continued conversations about *SDHA*-specific care.

Hypothesis: We hypothesize that the *SDHA*-associated tumor incidence in our cohort will be consistent with prior research indicating reduced penetrance compared with other hereditary PPGL genes, and that there is a corresponding lower clinical utility to standard high-risk screening protocols for unaffected *SDHA* carriers.

Objectives: We aim to describe the clinical characteristics, tumor spectrum, and surveillance outcomes of carriers with germline pathogenic/likely pathogenic *SDHA* variants identified at a single center from July 2015 to July 2025.

## Methods

This study includes both retrospectively identified tumor data and prospectively followed surveillance outcomes among patients attending the Family Cancer Assessment Clinic at the University of Utah Huntsman Cancer Institute. The clinic’s patient population primarily resides in the Intermountain West (Utah, Nevada, Wyoming, Idaho, and Montana) representing both rural and urban populations. Patients were identified through clinical genetic testing records and included those who were tested for an *SDHA* variant. Unaffected GPV carriers were recommended for surveillance, including annual biochemical screening (plasma or 24-h urine metanephrines) and imaging every 3–5 years. Imaging surveillance used FDG PET/CT for skull base to pelvis evaluation in most cases, supplemented by targeted CT and MRI studies. Screening outcomes were documented throughout the observation period. Tumor diagnoses and characteristics were verified through medical record review using Epic.

This study used data from December 2010 to July 2025. The analytic sample included 106 patients who had an *SDHA* GPV. Patients who had a variant of uncertain significance (VUS) and an *SDHA*-associated tumor history were excluded from the analytic sample (*n* = 4) and noted in Supplemental Table A.2 (see section on Supplementary materials given at the end of the article). The study was approved by the University of Utah Institutional Review Board (protocol #46740). The requirement for informed consent was waived by the IRB given the retrospective nature of the study and use of de-identified data. The study was conducted in accordance with the Declaration of Helsinki. All data were de-identified prior to analysis.

Demographic characteristics were self-reported, which include age at the time of genetic testing (Years), sex assigned at birth (Male, Female), race (White, Asian), and ethnicity (Hispanic, Non-Hispanic). Testing indication was a categorical measure determined by the carrier’s personal and family history at the time of genetic testing. Genetic test result was based on identification of a pathogenic or likely pathogenic *SDHA* variant in the genetic test report in their medical records. Classifications included an affected proband (first known person in the family with P/LP *SDHA* variant identified), cascade testing (*SDHA* testing due to a known familial variant), or secondary finding (multigene panel testing due to non-*SDHA*-related personal or family history).

Variant classification and type were defined by clinical genetic testing reports, which apply guidelines from the American College of Medical Genetics and Genomics (ACMG) and the Association for Molecular Pathology (AMP). These guidelines are subject to change over time as additional evidence becomes available. Variant classifications included likely pathogenic (LP), pathogenic (P), and variant of uncertain significance (VUS). Variant types included missense, nonsense, frameshift, multi-exon deletion, and splice-site.

Positive personal history (PHx) was defined as at least one diagnosis of an *SDHA*-associated tumor. Positive family history (FHx) was defined as a first-, second-, or third-degree blood relative diagnosed with an *SDHA*-associated tumor, on the same side of the family as the *SDHA* variant. Tumor diagnosis was a categorical measure of *SDHA*-associated tumors which included pheochromocytomas and paragangliomas (PPGLs), gastrointestinal stromal tumors (GISTs), and SDH-deficient renal cell carcinomas (RCCs). RCC cases were only included if SDHB immunohistochemistry (IHC) demonstrated loss of expression or if clinical context suggested SDH deficiency when IHC was unavailable. All other RCCs with non-SDH-related features were excluded. Tumor characteristics included tumor pathology and location, age at diagnosis, behavior (non-metastatic versus metastatic or malignant behavior), SDHB IHC status, and whether PPGLs were functional (catecholamine-secreting). Malignant disease was defined as a tumor with local or distant metastases identified on imaging or pathology. All other tumors were defined as benign with malignant potential. Clinically relevant abnormal metanephrine levels were defined as at least two times the upper limit of normal for that test.

Tables 1 and 2 provide the distribution of demographic and genetic characteristics of the analytic sample. Summary tumor diagnoses and characteristics are displayed in Table 3; expanded tumor information is displayed in Table 4. We used a contingency table to determine the appropriate statistical test to assess the relationship between variant type and positive PHx (Supplemental Table A.1). Due to low cell counts within the positive PHx column (less than 5), we used Fisher’s exact test, with an alpha decision factor of 0.05 (*P* < 0.05).

**Table 1:** Demographic and variant characteristics of *SDHA* pathogenic or likely pathogenic variant carriers*.

Characteristics	Total (*n* = 106)
Age at genetic testing (year), median (IQR)^†^	46 (28.3–58.0)
Biological sex (female), *n* (%)^‡^	67 (63.2)
Race, *n* (%)	
White	59 (55.7)
Asian	1 (0.9)
Not reported	46 (43.4)
Ethnicity (non-Hispanic), *n* (%)	51 (48.1)
Not reported	54 (50.9)
Variant classification, *n* (%)	
Pathogenic	84 (79.2)
Likely pathogenic	22 (20.8)
Variant type, *n* (%)	
Nonsense	53 (50.0)
Missense	28 (26.4)
Frameshift	5 (4.7)
Splice site	13 (12.3)
Multi-exon deletion	7 (6.6)
Attended hereditary paraganglioma clinic (yes), *n* (%)	56 (52.8)

* Data represent pathogenic (P) and likely pathogenic (LP) SDHA variant carriers identified through genetic testing. Not reported includes patients who declined to provide information or for whom data were unavailable.

^†^ Patient’s age at genetic testing ranged from 9 to 90 years.

^‡^ Biological sex is defined as the patient’s assigned sex at birth, categorized as female or male.

**Table 2:** Cohort classification by personal and family history of SDHA-associated tumors.

PHx/FHx status*	Testing indication^†^	*n* (%)^‡^	Hereditary PPGL clinic attendance rate (%)^§^
PHx+/FHx+	Affected proband	0 (0.0)	-
PHx+/FHx-	Affected proband	15 (14.1)	8/12 (66.7)
PHx-/FHx+	Cascade testing	23 (21.7)	12/21 (57.1)
PHx-/FHx-	Secondary finding	44 (41.5)	30/38 (78.9)
PHx-/FHx-	Cascade testing	24 (22.6)	14/24 (58.3)

SDHA, succinate dehydrogenase complex flavoprotein subunit A.

* PHx/FHx status is defined by having a positive or negative personal history (PHx) or family history (FHx) of an SDHA-associated tumor.

^†^ Testing indication was defined as an affected proband (first known person to have P/LP SDHA variants), cascade testing (SDHA testing due to a known familial variant), or secondary finding (multigene panel testing due to other non-SDHA-related personal or family history).

^‡^ Zero additional SDHA-associated tumors were found in PHx+ or PHx- carriers during the study period.

^§^ Rate of attendance refers to the proportion of patients who had at least one appointment at the hereditary PPGL clinic out of those recommended to do so.

**Table 3:** Summary statistics for affected carriers.

Characteristic	All tumors	Head/neck PGL	Extra-adrenal PGL	GIST
Total number	15	7	3	5
Median age at dx (range)	36 (17–69)	36 (24–69)	41 (17–45)	36 (17–62)
Biological sex (female), *n* (%)	4 (26.7)	1 (14.3)	1 (33.3)	2 (40.0)
Malignant, *n* (%)	7 (46.7)	-	3 (100)	4 (80.0)
Functional, *n*/*n* assessed (%)	1/10 (10.0)	-	1/3 (33.3)	-
SDHB IHC deficient, *n*/*n* tested (%)	7/9 (77.8)	3/4 (75.0)	1/1 (100)	3/4 (75.0)
Screening adherence, *n*				
Fully adherent	7	4	1	2
Partially adherent	2	1	-	1
Active cancer treatment	3	-	1	2
Discontinued – age	1	1	-	-
Lost to follow-up	2	1	-	-
Deceased	-	-	1	-

**Table 4:** Clinical characteristics of carriers with pathogenic or likely pathogenic *SDHA* variants and *SDHA*-associated tumors*.

*n*	Sex	Type	Location	Dx age	Current age	Tumor behavior	SDHB IHC	Functional^†^	Variant^‡^
1	F	PGL	Carotid body	24	30	Benign with MP	-	No	Ex1_3del
2	M	PGL	Carotid	22	26	Benign with MP	Deficient	No	c.91C>T
3	M	PGL	Vagal	28	38	Benign with MP	Intact	No	c.91C>T
4	M	PGL	Carotid	36	39	Benign with MP	Deficient	No	c.284C>G
5	M	PGL	Carotid body	36	39	Benign with MP	-	No	c.1663 + 1G>T
6	M	PGL	Carotid body	40	75	Benign with MP	-	No	c.91C>T
7	M	PGL	Vagal	69	71	Benign with MP	-	No	c.91C>T
8	F	PGL	Upper thoracic paraspinal	17	23	Malignant	Deficient	Yes	c.91C>T
9	M	PGL	Bladder	41	45	Malignant	-	No	c.91C>T
10	M	PGL	Retroperitoneal	45	50	Malignant	Deficient	No	c.1304T>G
11	M	GIST	Stomach	17	18	Benign with MP	Deficient	-	c.512G>A
12	F	GIST	Stomach	28	47	Malignant	Deficient	-	c.512G>A
13	F	GIST	Stomach	36	48	Malignant	Deficient	-	c.1471G>T
14	M	GIST	Stomach	41	46	Malignant	Intact	-	c.91C>T
15	M	GIST	Stomach	62	65	Malignant	-	-	c.91C>T

Dx, diagnosis; GIST, gastrointestinal stromal tumor; IHC, immunohistochemistry; LP, likely pathogenic; P, pathogenic; PGL, paraganglioma; SDHB, succinate dehydrogenase complex iron sulfur subunit B; Benign with MP, benign with malignant potential.

* No affected carriers were found to have multiple SDHA-associated tumors during the study period.

^†^ Functional refers to catecholamine-secreting PPGLs based on elevated metanephrines or normetanephrines via biochemical testing.

^‡^ SDHA variants reported using Mane Select NM_004168.4.

## Results

**Figure 1:**
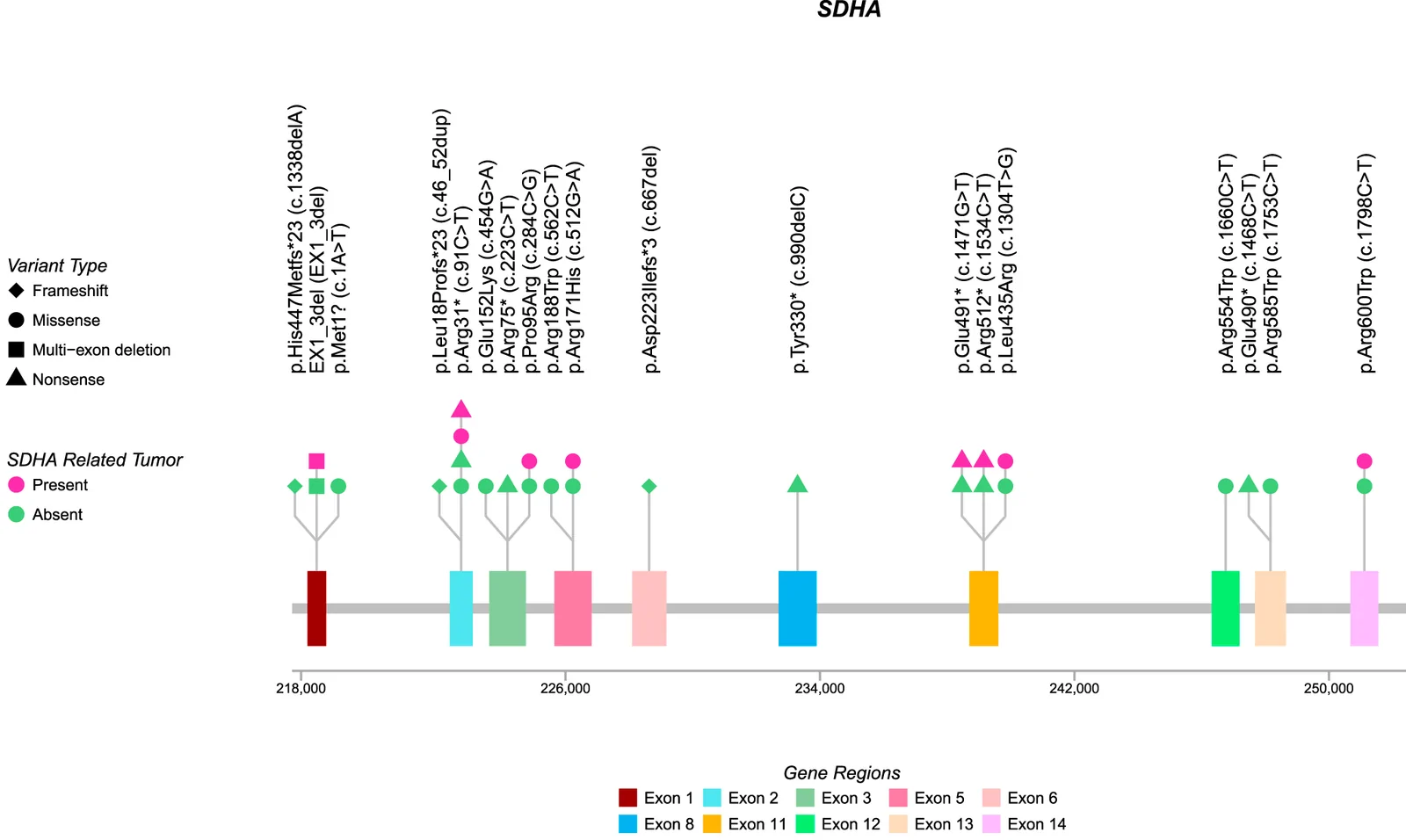
Location of identified pathogenic or likely pathogenic SDHA variants.

In the analytic sample, patients had a median age of 46 years with the majority identifying as white, female, and non-Hispanic. Fifty-six *SDHA* GPV carriers were seen for a new patient visit in the hereditary paraganglioma clinic at the Huntsman Cancer Institute (Table 1). Of the 106 patients with an *SDHA* GPV, 15.1% were probands with a positive PHx and negative FHx, 40.6% had secondary findings with a negative PHx and negative FHx, 21.7% had cascade testing with negative PHx and a positive FHx, and 22.6% had cascade testing with both a negative PHx and FHx. There were zero *SDHA* GPV carriers with both a positive PHx and FHx (Table 2). Of the patients who had hereditary paraganglioma clinic attendance recommended, the attendance rate was 78.9% of probands with negative PHx and negative FHx who had their SDHA variant identified as a secondary finding on multigene panel testing, 66.7% of probands with a positive PHx and negative FHx, 58.3% of those who had cascade testing with negative PHx and negative FHx, and 57.1% of those who had cascade testing for a known familial variant with negative PHx and positive FHx. There was no significant evidence of an association between variant type and personal history of an SDHA-associated tumor (*P* > 0.8). Recurrent variants included p.Arg31* (*n* = 36), p.Arg585Trp (*n* = 7), p.Glu491* (*n* = 6), p.Arg171His (*n* = 6), p.Arg75* (*n* = 6), p.Arg512* (*n* = 4), and p.Leu435Arg (*n* = 4) (Fig. 1).

**Figure 2:**
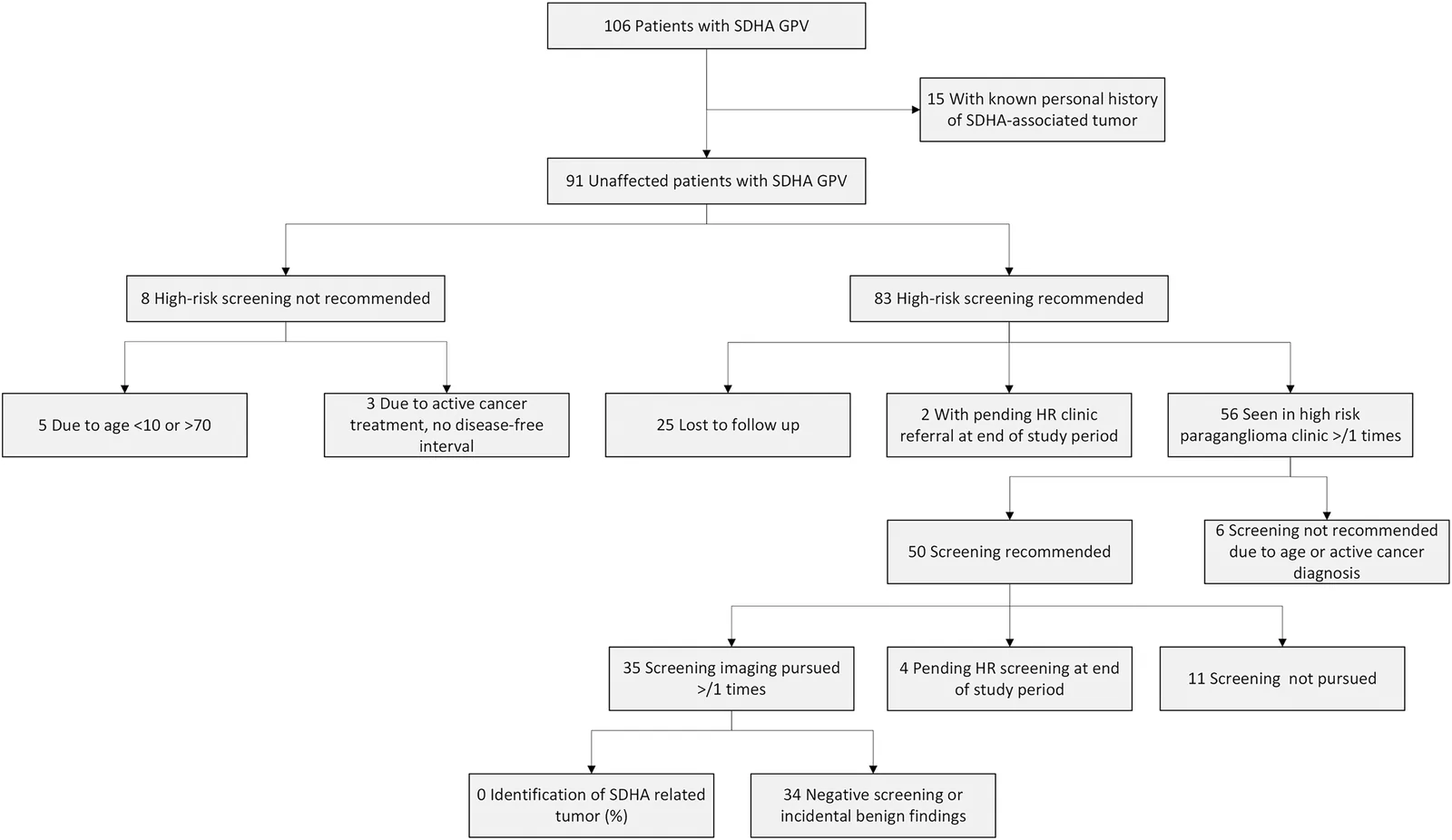
Clinical workup and outcomes for unaffected SDHA carriers at Huntsman Cancer Institute hereditary PPGL clinic.

Fifteen GPV carriers had a diagnosis of an *SDHA*-associated tumor, and the median age at tumor diagnosis was 36 years (Table 4). Tumor types were seven head/neck paragangliomas, three extra-adrenal paragangliomas, and five GISTs. None of these affected carriers had a known second primary SDHA-associated tumor identified during the study period. Of the tumors, 46.7% had malignant features; all extra-adrenal paragangliomas were malignant, and 80.0% of GISTs were malignant. Zero head/neck paragangliomas exhibited malignant features. Functional PPGLs were uncommon with only one among extra-adrenal PGLs. SDHB IHC showed absence or deficiency of SDHB in 80.0% of PPGLs and 75.0% of GISTs. Based on the available data, one of the 15 affected carriers was lost to follow-up during the study period, and two were partially adherent to recommended screening intervals. Eleven of the 15 were either fully adherent to recommended screening intervals (*n* = 7), discontinued screening due to age (*n* = 1), passed away (*n* = 1), or were in active cancer treatment and so screening and surveillance were pursued in conjunction with their cancer treatment (*n* = 3) (Table 4).

Of the 83 GPV carriers with a negative PHx for whom surveillance was recommended, 56 were seen in the hereditary paraganglioma clinic at least once and 35 had completed at least one screening imaging study by the end of the observation period (Fig. 2). Overall, 70% of unaffected patients who had screening recommended completed at least baseline imaging. Between December 2010 and July 2025, a total of 49 screening imaging studies were performed, of which 42 were completed baseline imaging (Supplementary Table A.3). FDG PET/CT (*n* = 33) was predominantly used for comprehensive skull base to pelvis evaluation, supplemented by targeted CT (*n* = 8) and MRI (*n* = 8) studies (Supplementary Table A.3). Imaging surveillance identified zero new *SDHA*-associated tumors in unaffected carriers. Follow-up imaging in previously affected probands only reidentified known tumors. Nine carriers (21% of those screened) had incidental benign findings identified on baseline surveillance imaging, eight of which required subsequent additional imaging or testing to confirm benign pathology. Forty-eight underwent baseline biochemical screening with plasma or 24-h urine metanephrines, and there were zero cases of clinically relevant abnormal metanephrines (Supplementary Table A.3).

## Discussion

As the work to develop gene-specific hereditary PPGL screening recommendations continues, our data show that long-term high-risk screening of this *SDHA* carrier population did not identify any previously unknown *SDHA*-associated tumors. This highlights the importance of moving toward gene-specific guidelines for *SDHA* carriers. The relatively low tumor incidence in our study cohort alongside a relatively high rate of malignant disease in affected carriers, driven primarily by extra-adrenal PPGLs and GISTs rather than head/neck PGL, is also consistent with prior studies of *SDHA* and other hereditary PPGL predisposition genes. This means that while tumor risks for *SDHA* carriers are low, clinical course and tumor characteristics for affected carriers differ from sporadic PPGLs and GISTs. These findings highlight a clinically important paradox: *SDHA* variants demonstrate low penetrance yet disproportionately aggressive tumor behavior when disease occurs, creating a challenge in balancing surveillance intensity with clinical utility. While it may not be unreasonable for unaffected *SDHA* GPV carriers to forego high-risk screening, patient education, shared decision-making, and informed discussions are critical. Also important are current and future research studies to better understand how factors such as family history or variant type may stratify *SDHA* penetrance to further tailor screening recommendations within this population.

### Screening outcomes

Our analysis of long-term outcomes of screening for *SDHA* carriers over a period of approximately 10 years found zero carriers with both a personal and a known family history of an *SDHA*-associated tumor; in fact, approximately 63% of *SDHA* carriers had no known personal or family history of a related tumor. This means that most carriers undergoing screening at our clinic were doing so due to the *SDHA* GPV finding alone. Other analyses of *SDHA* GPV cohorts have similarly found few cases where a GPV was inherited from an affected parent or where there was known family history of *SDHA*-associated tumors, with one pooled analysis identifying a related family history in only 4% of *SDHA* carriers (6, 9, 23). While the youngest age at diagnosis in our cohort was 17, PPGLs/GISTs have been identified in *SDHA* carriers at younger ages, including one report of an affected 8-year-old (24).

The identification of *SDHA* GPV carriers with related tumors and absent SDHB IHC in our cohort and others means that, at some point, screening unaffected *SDHA* carriers would be expected to identify a previously unknown related tumor (9, 25). However, in our cohort, long-term screening identified zero previously unidentified *SDHA*-associated tumors and zero carriers confirmed with multiple *SDHA*-associated tumors. Taken together with the absence of related family history in unaffected carriers, these screening outcomes suggest that approaching care for carriers with an *SDHA* GPV in the same way as other PPGL predisposition genes may result in overscreening of this cohort.

We also found that 21% of carriers who underwent imaging had ultimately benign incidental findings identified, most of which needed additional workup to confirm pathology. Prior research in a broader population undergoing whole-body MRI screening has found that disclosure of incidental findings on whole-body imaging resulted in moderate-to-severe psychological distress in close to a third of carriers (26). While attitudes about the screening process and results have not been analyzed for our higher-risk population, our data about screening outcomes support a nuanced approach to SDHA carrier screening. Screening for this population could incorporate recognition of its potential emotional and financial burden as part of patient education and decision-making, particularly given that ongoing surveillance is currently recommended from childhood through old age in most current guidelines (26, 27).

Overall, the results of our study seem to align with some of the conclusions from the 2023 UK guidelines for *SDHA* carriers, which recommend viewing *SDHA* GPVs in a carrier without a personal or family history of an *SDHA*-associated tumor as an incidental finding that does not necessitate ongoing screening (17). If our clinic had utilized those guidelines for our screening protocol, no cases of *SDHA*-associated tumors would have been missed. Usage of family history as a framework for risk stratification is consistent with current understanding of how family history can play a role in assessing cancer risk (28). It is well established that a family history of a tumor or cancer indicates a carrier’s personal risk is likely increased beyond what would be estimated based on the presence of a causative GPV alone. This is due to other risk factors that family members often share, such as environmental, lifestyle, and other currently unknown genetic factors (28). Although non-GPV risk factors for *SDHA* and other hereditary PPGL genes have not yet been identified, we lack evidence to indicate that this established model of cancer risk stratification does not hold true for this population.

However, data to specifically support family history of an *SDHA*-associated tumor as a proxy to designate someone at higher risk are limited. Our study did not find any families where the *SDHA* variant seemed to be more penetrant or any significant associations between variant type and tumor development. Additional research to better understand these factors and whether family history does indicate increased tumor risk for *SDHA* carriers will be important to ensure accurate risk assessment for this patient population.

### *SDHA*-associated tumor characteristics

The locations and types of *SDHA*-associated tumors seen in our cohort (seven head/neck paragangliomas, five GISTs, and three extra-adrenal paragangliomas) were largely consistent with those seen in prior studies. Other research has generally found that head/neck paragangliomas tend to be the most frequently seen *SDHA*-associated tumor, with adrenal and extra-adrenal paragangliomas and GISTs also commonly seen (9, 23, 24, 25, 29). One difference between our cohort and others is that we did not identify any carriers with multiple synchronous or metachronous *SDHA*-associated tumors, while they have been identified in rare cases in other *SDHA* GPV cohorts (9, 23).

Our finding of malignant disease in approximately 47% (*n* = 15) of affected carriers in our cohort with zero cases of malignant head/neck paraganglioma is also consistent with prior research (9). Other studies have found that *SDHA*-associated tumors may behave more similarly to *SDHB*-associated tumors, which are widely known to have increased malignancy risk, particularly for chest and abdominal PPGLs, compared with sporadic disease and tumors in the other SDHx complex genes *SDHC* and *SDHD* (9, 21, 24, 30, 31, 32). Our cohort data, derived from a clinical setting that does not primarily act as a referral center for carriers with severe or metastatic disease, independently support this characterization of *SDHA*-associated tumors. In addition, we did not find any significant association between variant type and *SDHA*-associated tumor history in our relatively small cohort of affected individuals. While to our knowledge variant type and penetrance have not previously been investigated for *SDHA*, a prior study focused on *SDHB* GPVs did find that truncating variants were significantly more common in affected individuals (33). For the time being, variant type or location continues to be unable to inform penetrance estimates for *SDHA*. Our finding of two cases of present/normal SDHB immunohistochemistry in affected carriers supports germline testing of all cases of PPGLs and wild-type GISTs, even when SDHB IHC is present.

### Implications

These findings support recent publications suggesting a nuanced, individualized approach to *SDHA* carrier screening instead of applying the intensive surveillance protocols used for other *SDH* gene variants. For carriers without a personal or family history of *SDHA*-associated tumors in particular, the relatively low lifetime tumor risk and the lack of screening utility seen in our cohort and others suggest that the financial, emotional, and medical downsides to intensive lifelong screening should be weighed alongside the benefits, and it may be reasonable for these carriers to pursue modified screening intervals or to abstain from formal surveillance.

Our study data support this view, but uniformly enacting it in real-life clinical care may prove challenging. A benefit of this approach is that it acknowledges the lack of data indicating how to stratify risk within the *SDHA*+ population and gives patients agency in deciding how much screening to pursue. Downsides, however, may include guidelines for providers that may lead to confusion about how to screen patients, particularly for those who rarely see *SDHA* carriers. This could result in increased confusion for both providers and patients. Additional resources for provider and patient education will be important if guidelines head in this direction to facilitate more equitable patient care for carriers not being seen at tertiary care centers well versed in this genetic predisposition.

Existing alongside this decision-making nuance is the systemic challenge of international coordination of hereditary PPGL screening protocols. Studies have collated expertise from international hereditary PPGL experts to generate consensus guidelines, acknowledging differences in cost of and access to specific screening modalities across healthcare systems (5, 19, 34). In countries with publicly funded and centralized healthcare systems, national screening programs may require formal evaluation such as cost-effectiveness analysis before clinical practice can shift, and the low lifetime tumor risk for *SDHA* carriers may make this analysis particularly challenging as our understanding of this gene continues to evolve (35, 36, 37).

Family history of an *SDHA*-associated tumor seems initially to be a reasonable method of risk stratification within this patient population, but additional research to clarify whether this is the case is another important future direction for the field. Continued research with larger datasets into whether variant type impacts disease penetrance is also needed to inform patient care. From a treatment perspective, the implications of *SDHA* germline variants extend beyond screening paradigms. Our findings of relatively low penetrance but high rates of malignant behavior among affected carriers suggest that identification of an *SDHA* variant may influence operative decision-making and threshold for surveillance versus intervention. Notably, no head and neck paragangliomas in our cohort demonstrated malignant behavior, consistent with prior literature suggesting lower metastatic potential in this subgroup. This distinction may further support a differentiated management strategy for SDHA-associated tumors based on anatomic site. Overall, *SDHA* status may represent an additional factor in risk stratification, particularly given the potential for aggressive behavior despite low overall incidence.

While there is a clear utility to knowing *SDHA* status in an affected individual, the low lifetime tumor risk raises the question of whether P/LP *SDHA* variants should be reported when found incidentally. *SDHA* is not on the ACMG secondary findings list, and 2023 UK *SDHA* guidelines have proposed treating some *SDHA* findings as non-actionable secondary results (17, 38, 39). However, removing *SDHA* from standard multigene panels would require ordering providers to independently recognize *SDHA*-associated tumors and request targeted testing. This may be unrealistic given low provider confidence in navigating genetic test selection (40, 41). In the United States, insurance denials for genetic testing remain common, and obtaining coverage for a second test is often more difficult than including additional genes for a minimal marginal cost on an initial test (42, 43). Furthermore, incidental identification of *SDHA* carriers enables cascade testing of at-risk relatives who may have undiagnosed tumors with malignant potential. These practical considerations must be weighed against the potential harms of disclosing a variant with uncertain actionability due to its low lifetime risks, further reinforcing the need for robust patient and provider *SDHA* educational resources.

### Strengths and limitations

The main strength of our study is that we primarily serve patients who live within the region surrounding our cancer center (Utah, Idaho, Montana, Wyoming, and Nevada) and do not have a significant proportion of patients seeking PPGL care from further afield due to complex and/or metastatic disease. Therefore, our data are theoretically more representative of general population *SDHA* tumor characteristics, penetrance, and screening outcomes than a cohort drawn from a center that derives more of their patient population from non-local referrals. Our institution’s adoption of point-of-care multigene panel testing for a broad group of patients seen at Huntsman Cancer Institute for cancer care also helps to reduce self-selection bias. This bias comes from certain patient populations being more likely to opt-in to getting genetic testing than others. However, there are still discrepancies within our institution related to which provider specialties are universally adopting this testing process versus those who are not regularly testing all individuals with a PPGL for hereditary tumor predisposition.

Limitations include that our screening data cohort is a convenience sample of people seen in the high-risk PPGL clinic and may not be representative of the *SDHA* carrier cohort overall. In addition, we have limited socio-demographic data available about our patient population and so it may not be representative of the *SDHA* GPV carrier population as a whole. This limits the generalizability of our findings to a degree and means that the *SDHA* variants identified in our study may not be reflective of broader population prevalence.

Another limitation is that some carriers in our cohort have been lost to follow-up during the study period, including two affected carriers, in which case any development of additional tumors or metastatic disease would not be captured by our data, and tumor risks and metastasis rates could be underrepresented. At our institution, carriers with an *SDHA*-associated tumor are not typically followed by the high-risk paraganglioma clinic during treatment but are primarily managed by the specialty most relevant to their tumor type, location, and characteristics until treatment and surveillance is complete. Since many affected *SDHA* carriers in our cohort have metastatic disease, their ongoing screening and surveillance have been managed through neuroendocrine tumor oncology with ongoing coordination with our clinic for other services such as family variant testing. This means that while these carriers are getting ongoing imaging and lab work related to both their metastatic disease and their *SDHA* variant, modified screening intervals and/or modalities may be used since cancer treatment typically has superseded high-risk screening protocols.

In addition, this study did not incorporate data from individuals in our cohort with an *SDHA*-associated tumor and an *SDHA* variant of uncertain significance, given the potential for a coincidental benign *SDHA* variant in someone with a sporadic tumor. However, it is likely that future variant reclassifications will clarify that some of these variants are pathogenic. To assist with the development of future research, we provided clinical and genetic classifications for the four VUS patients that were excluded from our study (Supplementary Table A.2).

## Conclusion

This research study provides additional impetus for healthcare providers who care for PPGL, GIST, and *SDHA*+ patients to consider how *SDHA* screening guidelines can be tailored to more realistically reflect the paradox of GPVs in this gene. As multigene panel testing becomes more commonplace as a standard component of cancer treatment, we expect the number of *SDHA* GPV carriers without related history to grow (44, 45, 46). Our data about affected carriers and screening outcomes over a period of 10 years demonstrate that following a ‘one-size-fits-all’ approach to hereditary PPGL screening likely results in overscreening of this population, given the many decades of screening that patients undergo based on current guidelines. However, due to the nontrivial risk of malignant disease, if a tumor develops, an *SDHA*+ test result is not a straightforward incidental finding. Patient education about risks and symptoms is important at baseline, as is shared decision-making about proceeding with screening given the benefits and drawbacks. Knowing whether someone with a related tumor is *SDHA*+ can also inform treatment decisions. These include proceeding with surgery versus tumor surveillance, preoperative imaging to identify additional tumors or any metastasis, and the duration/frequency of subsequent surveillance post-surgery given malignancy risks.

## Supplementary materials



## Declaration of interest

There is no conflict of interest that could be perceived as prejudicing the impartiality of the research reported.

## Funding

This work was supported in part by the National Cancer Institute Cancer Center Support Grant P30CA042014 (Huntsman Cancer Institute). No additional funding was received for this study.

## Author contribution statement

KE wrote and revised the paper and performed data analysis. DC wrote and revised the paper and performed data analysis. JB performed data analysis. AN contributed to conception of the paper, acquired data, and reviewed it. JV, RW, and LB contributed to conception of the paper and reviewed it. HM reviewed the paper
