# Supplementary material for: Tumor risks and surveillance outcomes in SDHA variant carriers
Source: Endocr Oncol. 2026 Sep 11;6(1):e260048. doi: 10.1530/EO-26-0048 (PMC13588288; doi:10.1530/EO-26-0048)
Supplement: Supplementary file 1 [file EO-26-0048_supplementary_tables.pdf]

SUPPLEMENTARY MATERIAL

Supplemental Table A.1: Descriptive Contingency Table of Variant Type and Personal History of an SDHA-Related Tumor

Variant Type	Positive PHx	Negative PHx	Total
Missense	5	23	**28**
Nonsense	8	45	**53**
Frameshift	0	5	**5**
Multi-exon Deletion	1	6	**7**
Splice Site	1	12	**13**
**Total**	**15**	**91**	**106**

Supplemental Table A.2: Clinical Characteristics of Carriers With SDHA Variants of Uncertain Significance and SDHA-Associated Tumors

n	Sex	Type	Location	Dx Age	Current Age	Behavior	SDHB IHC	Funct-ional	Variant
1	F	PGL	Vagal	24	32	Benign with MP	Not done	No	c.1055G>A
2	M	PGL	Cerebellop-ontine angle	49	51	Benign with MP	Not done	No	c.106A>G
3	M	PGL	Carotid body	70	83	Benign with MP	Not done	No	c.106A>G
4	M	PGL	Carotid body	72	75	Benign with MP	Intact	No	c.305C>T

Supplemental Table A.3: Additional Biochemical Screenings, Imaging Surveillance, and Tumor Characteristics

**Characteristics**	**Count**	**Percentage**
**Biochemical Screening**		
Baseline screening completed (Yes)	48/90	53.3%
Any biochemical screening (Yes)	51/90	56.7%
Clinically relevant abnormal metanephrines (>2)	0/48	0%
**Imaging Surveillance**		
Baseline imaging completed	42/90	46.7%
Total screening scans performed	49	—
Imaging Modality		
FDG PET/CT	33	67.3%
CT scan (chest/other)	5	10.2%
MRI Abdomen	3	6.1%
CT Chest	3	6.1%
MRI Neck	3	6.1%
Other MRI (Pelvis, Whole Body)	2	4.1%
**Tumor Detection**		
New SDHA-associated tumors identified through screening	0	
No findings/normal/stable results	34	
Incidental Finding after screening		
Total incidental benign findings	9	
Required additional imaging/testing for confirmation	8	
Simple observation only	1	
